# Cardiovascular Autonomic Nervous System Function and Aerobic Capacity in Type 1 Diabetes

**DOI:** 10.3389/fphys.2012.00356

**Published:** 2012-09-07

**Authors:** Harriet Hägglund, Arja Uusitalo, Juha E. Peltonen, Anne S. Koponen, Jyrki Aho, Suvi Tiinanen, Tapio Seppänen, Mikko Tulppo, Heikki O. Tikkanen

**Affiliations:** ^1^Department of Sports and Exercise Medicine, Institute of Clinical Medicine, University of HelsinkiHelsinki, Finland; ^2^Foundation for Sports and Exercise MedicineHelsinki, Finland; ^3^Department of Electrical and Information Engineering, University of OuluOulu, Finland; ^4^Verve ResearchOulu, Finland

**Keywords:** aerobic capacity, alpha1, detrended fluctuation analysis, heart rate variability, type 1 diabetes

## Abstract

Impaired cardiovascular autonomic nervous system (ANS) function has been reported in type 1 diabetes (T1D) patients. ANS function, evaluated by heart rate variability (HRV), systolic blood pressure variability (SBPV), and baroreflex sensitivity (BRS), has been linked to aerobic capacity (VO_2peak_) in healthy subjects, but this relationship is unknown in T1D. We examined cardiovascular ANS function at rest and during function tests, and its relations to VO_2peak_ in T1D individuals. Ten T1D patients (34 ± 7 years) and 11 healthy control (CON; 31 ± 6 years) age and leisure-time physical activity-matched men were studied. ANS function was recorded at rest and during active standing and handgrip. Determination of VO_2peak_ was obtained with a graded cycle ergometer test. During ANS recordings SBPV, BRS, and resting HRV did not differ between groups, but alpha1 responses to maneuvers in detrended fluctuation analyses were smaller in T1D (active standing; 32%, handgrip; 20%, medians) than in CON (active standing; 71%, handgrip; 54%, *p* < 0.05). VO_2peak_ was lower in T1D (36 ± 4 ml kg^−1^ min^−1^) than in CON (45 ± 9 ml kg^−1^ min^−1^, *p* < 0.05). Resting HRV measures, RMSSD, HF, and SD1 correlated with VO_2peak_ in CON (*p* < 0.05) and when analyzing groups together. These results suggest that T1D had lower VO_2peak_, weaker HRV response to maneuvers, but not impaired cardiovascular ANS function at rest compared with CON. Resting parasympathetic cardiac activity correlated with VO_2peak_ in CON but not in T1D. Detrended fluctuation analysis could be a sensitive detector of changes in cardiac ANS function in T1D.

## Introduction

The autonomic nervous system (ANS) is a major regulator of the cardiovascular system. It regulates heart rate and blood pressure in the short-term to cope with everyday situations. Parasympathetic (vagal) modulation decreases the heart rate and cardiac contractility, whereas activity of the sympathetic branch opposes these effects and regulates peripheral vasoconstriction.

Balanced cardiac ANS function is based on strong parasympathetic and efficient, but not overactive, sympathetic modulation of the heart. Efficient ANS function has been associated with lower risk of cardiovascular complications (Huikuri et al., 2000). The relationships between aerobic exercise training or high aerobic capacity and enhanced vagal modulation of the heart have been widely reported, especially in healthy populations (Billman, 2002; Buch et al., 2002; Carter et al., 2003; Hautala et al., 2009). Also the relation between exercise and decreased sympathetic activity has been suggested (Billman, 2002). Impaired cardiovascular ANS function has been associated with type 1 diabetes (T1D) (Ziegler et al., 2001; Riihimaa et al., 2002; Dalla Pozza et al., 2007; Lucini et al., 2009; Rosengård-Bärlund et al., 2009; Spallone et al., 2011; Kuehl and Stevens, 2012). Recently, we reported lower aerobic capacity (VO_2peak_) in Type 1 diabetes patients than in healthy controls (Peltonen et al., 2012), which is in agreement with several other papers (Niranjan et al., 1997; Komatsu et al., 2005; Gusso et al., 2008; Nadeau et al., 2010). However, the associations between ANS function and aerobic capacity are not as well known in T1D than in several other populations.

Impaired cardiovascular ANS function (Manzella and Paolisso, 2005; Carnethon and Craft, 2008) and lower aerobic capacity (Albright et al., 2000) are often related also to type 2 diabetes. The suggested possibly mechanisms to diabetic autonomic dysfunction include hyperglycemia induced ANS damages and effects of hyperinsulinemia due to insulin resistance (Manzella and Paolisso, 2005; Carnethon and Craft, 2008). T1D is caused by a relative or absolute deficiency of insulin secretion, but type 2 diabetes is mainly characterized as an insulin resistance state. Although this division is not strict, as insulin resistance can also exist in T1D and insulin deficiency in type 2 diabetes, the etiology of the diseases differs greatly. In addition, physical activity has been inversely associated with onset of type 2 diabetes also independently of weight loss (Bassuk and Manson, 2005; Carnethon and Craft, 2008). This may be due to improved insulin sensitivity or effects on ANS function, or both. In addition, exercise training has also been related to improved cardiovascular ANS function in patients who already have caught type 2 diabetes (Loimaala et al., 2003; Pagkalos et al., 2008). Similar associations between exercise, cardiovascular ANS function, and disease itself are not as well known in T1D, and they cannot be generalized between type 1 and type 2 diabetes. The further knowledge of these relations is needed, when planning and developing the exercise training instructions and treatment recommendations to T1D patients.

Quantification of cardiovascular ANS function has widely been performed with supine rest recording and with deep breathing, active standing, and handgrip tests (Ewing et al., 1985). Combined with the sophisticated modern analyses of heart rate variability (HRV), systolic blood pressure variability (SBPV), and baroreflex sensitivity (BRS), changes in cardiovascular ANS function of T1D patients can be detected before any manifestation of clinical neuropathy symptoms (Task Force of ESC and NASPE, 1996; Weston et al., 1996, 1998; Ziegler et al., 2001; Spallone et al., 2011; Kuehl and Stevens, 2012). Accordingly, T1D-related changes in cardiovascular ANS function have been reported in adults (Weston et al., 1996, 1998; Ziegler et al., 2001; Rosengård-Bärlund et al., 2009), as well as in adolescents and children (Riihimaa et al., 2002; Dalla Pozza et al., 2007; Lucini et al., 2009). In addition, the studies are diverse concerning maneuvers and ANS variables. In some studies, cardiovascular ANS function changes in T1D patients were found in supine (Weston et al., 1998; Dalla Pozza et al., 2007; Lucini et al., 2009), but in others only in active standing (Ziegler et al., 2001; Riihimaa et al., 2002). The greatest impairments in T1D patients often have existed in BRS and SBPV (Ziegler et al., 2001; Dalla Pozza et al., 2007; Lucini et al., 2009). However, these previous studies do not contain data on aerobic capacity of the subjects. In addition, at least to our knowledge, detrended fluctuation analyses have not been reported in T1D patients. This non-linear HRV method reflects different phenomena than widely used time and frequency domain HRV methods (Peng et al., 1995).

The aim of this study was to examine if cardiovascular ANS function supine at rest and during deep breathing, active standing, and handgrip tests was related to T1D and to lower aerobic capacity in T1D patients than in healthy controls. In addition, we aimed at versatile profiling of cardiac ANS function differences between T1D patients and healthy controls (CON) by analyzing HRV by time domain, frequency domain, and non-linear methods. Our hypotheses were that cardiovascular ANS function would be altered in T1D patients relative to healthy and that non-linear methods might be sensitive to indicate these changes. In addition, we hypothesized that VO_2peak_ would associate with cardiovascular ANS function in T1D patients.

## Materials and Methods

### Subjects

Study subjects comprised 10 T1D patients and 11 CON. From these, nine T1D and nine CON were the same subjects as in our recent paper (Peltonen et al., 2012). All were non-smoking men without diagnosed cardiovascular, renal or psychiatric diseases, high blood pressure, abundant ectopic heart beats, or regular medication, except for insulin in T1D. The study groups did not differ in age or anthropometric data, and were matched by their leisure-time physical activity (LTPA; Table 1).

**Table 1:** **Characteristics of the study population and results**.

Characteristic	Type 1 diabetes patients	Controls
*n*	10	11
Age (years)	34 ± 7	31 ± 6
Duration of type 1 diabetes	11 ± 6	−
HbA1c (%)	7.7 ± 0.9^†^	−
Height (cm)	177.8 ± 8.8	183.7 ± 5.4
Weight (kg)	77.0 ± 11.5	85.1 ± 10.7
BMI (kg/m^2^)	24.4 ± 3.1	25.2 ± 3.3
Body fat (%)	15.5 ± 5.3	17.2 ± 8.3
LTPA (min/week)	289 ± 155	352 ± 156
VO_2peak_ (ml kg^−1^ min^−1^)	36 ± 4*	45 ± 9
HR_max_ (bpm)	180 ± 9	184 ± 14
30/15 ratio	1.61 ± 0.32	1.41 ± 0.30
max/min RRIs	1.28 ± 0.11^†^	1.32 ± 0.16

Values are means ± SD. HbA1c, glycosylated hemoglobin; BMI, body mass index; LTPA, leisure-time physical activity; HR_max_, maximum heart rate during exercise; bpm, beats per minute; 30/15 ratio, lying to standing heart rate response; max/min RRIs, maximum to minimum ratio of RRIs in deep breathing test. *Difference between groups; *p* < 0.05; ^†^*n* = 9.

### Experimental design

The protocol was in accordance with the Declaration of Helsinki and was approved by the Ethics Committee of the Hospital District of Helsinki. All subjects gave written informed consent. The aerobic capacity measurement and body composition analysis were performed on the subjects’ first visit to the laboratory and the cardiovascular ANS function testing on the second, so that subjects were familiar with a face mask for respiration frequency measurements before ANS function recordings. HbA_1C_ was analyzed from venous blood samples of T1D. Subjects answered written questions about LTPA. Subjects were asked to abstain from alcohol and vigorous exercise for 24 h and caffeine for 12 h before testing.

### Aerobic capacity measurement and body composition analysis

Body composition analysis was performed by using a bioimpedance method (Inbody 720, Biospace Co., Ltd., Seoul, South-Korea), and body mass index (BMI) was calculated. Before the cycle ergometer test, the physician evaluated the suitability of subjects for the maximal exercise test. Glucose concentration from a fingertip blood sample was controlled to be in the range of 5.6–16.7 mmol/l before and 10 min after the exercise test. The patients injected the insulin that they routinely use or used oral glucose supplementation if needed. The graded cycle ergometer test was preceded by a 6-min resting period with the subject sitting on the cycle ergometer and then a 5-min unloaded cycling period. The test protocol consisted of 40 W increments every 3 min until the subject’s voluntary fatigue. Ventilation and alveolar gas exchange were measured breath-by-breath by a low-resistance turbine (Triple V, Jaeger Mijnhardt, Bunnik, Netherlands) and a mass spectrometer (AMIS 2000, Innovision, Odense, Denmark). To obtain VO_2peak_ values, gas delay determinations were performed to measured raw breath-by-breath data, and then alveolar gas exchange was calculated with the slightly modified algorithms of Beaver et al. (1981). Moving averages of individual test data were calculated over 5 s periods to reduce breath-by-breath variability, and interpolated to obtain values second by second, after which a VO_2peak_ value was determined as the highest value of a 60-s moving average window.

### Autonomic nervous system function measurements

Autonomic nervous system function testing was performed in a quiet, dimly lit laboratory between 08:00 and 13:00 hours. The ANS function test battery included four different 5-min tests: (1) resting, (2) deep breathing (3 min × 1 min with the breathing frequency of 6/min), (3) active standing, and (4) handgrip, all of which were preceded by 5-min rest periods. Resting, deep breathing, and handgrip tests were performed while subjects were in a supine position. The subjects maintained a sustained handgrip with a hydraulic hand dynamometer (Baseline, Fabrication Enterprises, Inc., New York, NY, USA) at 30% of their maximum voluntary contraction, which was measured before the ANS function recording protocol. The subjects performed handgrip test with a 90° angle in their right elbow joint, and the arm was supported by a pillow. They received verbal instructions to hold right grip strength and saw the strength indicator of a dynamometer via a mirror. The handgrip test was terminated if the subject was unable to maintain the required force of contraction and fatigued before the 5-min mark.

Continuous beat-by-beat blood pressure from a left middle finger was recorded via plethysmography (Nexfin, Bmeye, Amsterdam, Netherlands). In addition, the signal of respiratory flow from a low-resistance turbine and a mass spectrometer together with a standard II-lead ECG signal were collected by a Powerlab data acquisition system (Powerlab, ADInstruments, Sydney, Australia) with a sampling frequency of 1,000 Hz.

### Signal analyses

Baroreflex sensitivity and time and frequency domain analyses of HRV and SBPV were performed with Barox (Oulu, Finland) and Powerlab 5.0 software, and non-linear HRV analyses with Kubios 2.0 software (Kuopio, Finland). Data were resampled at 2 Hz (Task Force of ESC and NASPE, 1996). ECG and blood pressure recordings were inspected visually, and the recordings with poor quality were excluded from the analyses. Some of the blood pressure recordings included automatic signal calibration during two or three heartbeats every 70th beat. These periods were interpolated based on three previous beats, as were done with ectopic heartbeats in ECG and blood pressure signals. Analyses were performed during the last 2 min of resting periods, active standing, and handgrip tests, to obtain the effects of each maneuver and during comparable time periods.

Spectral HRV, SBPV, and BRS parameters were analyzed as a low frequency (LF) component (0.04–0.15 Hz). The LF component of HRV reflects parasympathetic and sympathetic cardiac ANS modulation and is related to arterial blood pressure control (Task Force of ESC and NASPE, 1996). HRV was analyzed also as a high frequency (HF) component (0.15–0.4 Hz), which is considered to be an estimate of parasympathetic ANS activity based on mainly sinus arrhythmia (Task Force of ESC and NASPE, 1996). Spectral methods were performed with an autoregressive model (Burg’s algorithm), and the model order was determined by the Akaike information criterion. BRS was investigated by frequency domain analyses of SBPV and concomitant variability of heart rate using an alpha method (Pagani et al., 1988). BRS analyses with a coherence criterion of at least 0.5 were accepted. In addition, HRV time domain parameters, the square root of the mean of the sum of the squares of differences between adjacent RRIs (RMSSD) for parasympathetic activity and standard deviation of RRIs (SDNN) for total HRV were analyzed (Task Force of ESC and NASPE, 1996).

Also non-linear HRV analyses of Poincaré plot and detrended fluctuation analysis were performed. SD1 of Poincaré plot represents the standard deviation of the short-term RR interval variability origin from cardiac parasympathetic regulation (Brennan et al., 2001). Detrended fluctuation analysis and its parameter fractal scaling exponent alpha1 describes the short-term, in this study 4–11 beats, fractal correlation properties within the RRI data (Peng et al., 1995; Tulppo et al., 2001). Changes in alpha1 have been correlated with changes in the LF:HF ratio related to ANS function tests (Tulppo et al., 2005).

The 30/15 ratio of active standing was calculated as the ratio between the longest and the shortest RRI after standing up (Ewing et al., 1985). Maximum to minimum ratio of RRI (max/min RRI) during the breathing cycle was calculated as a mean of 12 cycles (four of each min). The “delta” values of variables were calculated by taking the value during the last 2 min of active standing or the handgrip test and subtracting the value during the preceding 2-min resting period. The percentage change caused by the maneuver was calculated by the delta value and by the non-transformed value of the preceding rest period.

### Statistics

Data were analyzed by PASW Statistics 17 (SPSS, Chicago, IL, USA). The normal distribution of the variables was tested by the Shapiro–Wilk goodness-of-fit test. Non-parametric statistics were used because of several non-Gaussian distributed variables. Differences between T1D and CON in analyzed variables were tested by using Mann–Whitney *U*-test. The effect of a maneuver was tested by comparing the value of the maneuver to the corresponding value of the preceding rest period by Wilcoxon signed-rank test. Correlations were tested by Spearman’s correlation. Significance was defined as *p* < 0.05. The results are presented as medians (interquartile range), if not indicated as means ± SD.

## Results

Type 1 diabetes patients had lower VO_2peak_ than CON, although the groups were matched by the amount of self-reported LTPA (Table 1). Aerobic capacity correlated with LTPA in CON (*r* = 0.66, *p* = 0.03), but not in T1D (*r* = −0.38, *p* = 0.28). Heart rate, blood pressure, breathing frequency, and time and frequency domain analyses of HRV and BRS did not differ between T1D and CON at rest or during active standing or the handgrip test (Tables 2 and 3). Absolute and percentage changes of ANS measures related to the maneuvers were also similar in both groups. LF variability of systolic blood pressure at rest was 8.7 (8.7) mmHg^2^ in T1D and 17.8 (13.0) mmHg^2^ in CON and failed to differ significantly between the groups (*p* = 0.06). During active standing, LF of SBPV increased in T1D [586 (744)%, *n* = 9] and in CON [123 (441)%, *n* = 11], but not during the handgrip test [T1D: 37 (130)%, *n* = 9; CON: 96 (211)%, *n* = 11] and no group differences were seen. Nor did the groups differ in 30/15 ratio of active standing or maximum to minimum ratio of deep breathing (Table 1).

**Table 2:** **Values of autonomic nervous system function variables at rest and changes in active standing and in the handgrip test compared with preceding rest period in T1D (type 1 diabetes patients) and CON (healthy controls)**.

	Rest		ΔActive standing		ΔHandgrip	
	T1D	CON	T1D	CON	T1D	CON
Heart rate (bpm)	59 (12)	58 (16)	+20* (13)	+26* (16)	+22* (16)^‡^	+27* (21)^†^
Breathing frequency (Hz)	0.19 (0.12)^‡^	0.20 (0.18)	+0.03* (0.03)^‡^	0.00 (0.08)	+0.08* (0.08)^‡^	+0.05*(0.11)
Blood pressure
Systolic (mmHg)	118 (18)	121 (22)	+4.4 (11)	−5.0 (24)	+40* (20)^§^	+41* (23)
Diastolic (mmHg)	66 (8)	65 (7)	+11* (9)	+7.6* (12)	+26* (16)^§^	+29* (9)

Values are medians (interquartile ranges).*Significant difference between the value of the last 2 min of the maneuver and that of the last 2 min of the preceding rest period within the group (*p* < 0.05). ^†^*n* = 10 (CON); ^‡^*n* = 9; §*n* = 8.

**Table 3:** **Values of heart rate variability measures and baroreflex sensitivity at rest and percentage changes in active standing and in the handgrip test compared with the preceding rest period in T1D (type 1 diabetes patients) and CON (healthy controls)**.

	Rest		Change in standing		Change in handgrip	
	T1D	CON	T1D	CON	T1D	CON
SDNN (ms)	61 (38)	77 (49)	−6% (43)	−16%^†^ (48)	−6% (54)	−24%^†^ (65)^‡^
RMSSD (ms)	42 (27)	69 (57)	−46%^†^ (32)	−32%^†^ (72)	−58%^†^ (44)^§^	−65%^†^ (29)^‡^
HF (ms^2^)	622 (1460)	1161 (1167)	−60%^†^ (64)	−85%^†^ (77)^‡^	−74%^†^ (39)^§^	−81%^†^ (32)^‡^
LF (ms^2^)	1820 (3417)	858 (3213)^‡^	−3% (67)^§^	+19% (141)	−81%^†^ (41)^§^	−82%^†^ (49)^‡^
LF:HF	2.5 (4.4)	2.1 (3.5)^‡^	+227%^†^ (575)^§^	+415%^†^ (1490)^‡^	+35% (267)^§^	+91% (276)^§^
DFA: alpha1	1.2 (0.6)	1.0 (0.4)^‡^	+32%*^†^ (39)	+71%^†^ (80)	+20%* (48)	+54%^†^ (69)^‡^
Poincaré: SD1 (ms)	30 (19)	42 (34)^‡^	−46%^†^ (32)	−63%^†^ (42)	−48%^†^ (41)	−66%^†^ (37)^‡^
Baroreflex sensitivity
LF (ms/mmHg)	14 (11)	15 (12)^§^	−63%^†^ (32)^§^	−53%^†^ (62)	−65%^†^ (44)^¶^	−70%^†^ (33)^Ⅱ^

Values are medians (interquartile ranges). RRI, RR interval; SDNN, SD of RRIs; RMSSD, square root of the mean of the sum of the squares of differences between adjacent RRIs; HF, high frequency; LF, low frequency; DFA, detrended fluctuation analysis; SD1, SD of the short-term RR interval variability. *Significant difference between the groups (*p* < 0.05); ^†^ significant difference between the value of the last 2 min of the maneuver and that of the last 2 min of the preceding rest period within the group (*p* < 0.05); ^‡^*n* = 10 (CON); ^§^*n* = 9; ^Ⅱ^*n* = 8; ^¶^*n* = 7.

**Figure 1:**
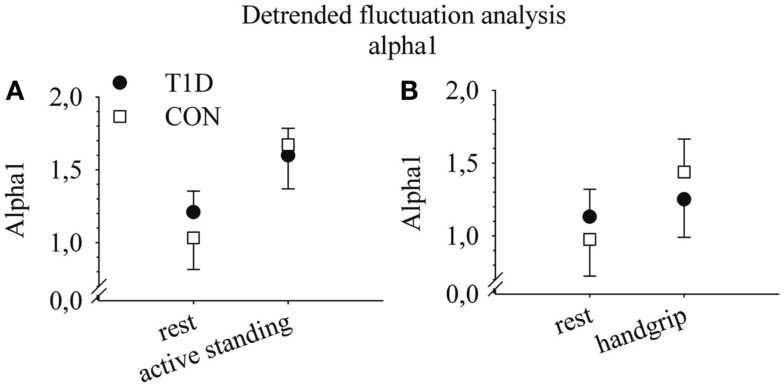
**Alpha1 values at rest and in active standing (A) and at rest and in the handgrip test (B) in type 1 diabetes patients (T1D) and in healthy controls (CON)**. Medians and quartiles 1 and 3. For the sake of clarity, only one other quartile is visible. *n* = 10 (T1D) and 11 (CON) in active standing and *n* = 10 (T1D) and *n* = 10 (CON) in the handgrip test.

The HRV measure of SD1 did not differ between T1D and CON. However, the percentage changes related to active standing and the handgrip test in the detrended fluctuation analysis variable alpha1 were smaller in T1D than in CON (Table 3; Figure 1). Because of the differences between T1D and CON in alpha1 responses to the maneuvers, the correlation between percentage alpha1 changes and VO_2peak_ was also tested. The percentage changes of alpha1 related to the handgrip test correlated with VO_2peak_ in CON and when the groups were analyzed together (*r* = 0.76, *p* = 0.01 and *r* = 0.66, *p* = 0.02, respectively), but not in T1D (*r* = 0.08, *p* = 0.83). The changes related to active standing did not correlate with VO_2peak_ (T1D: *r* = −0.30, *p* = 0.93; CON: *r* = 0.47, *p* = 0.14, and groups together: *r* = 0.44, *p* = 0.05).

**Figure 2:**
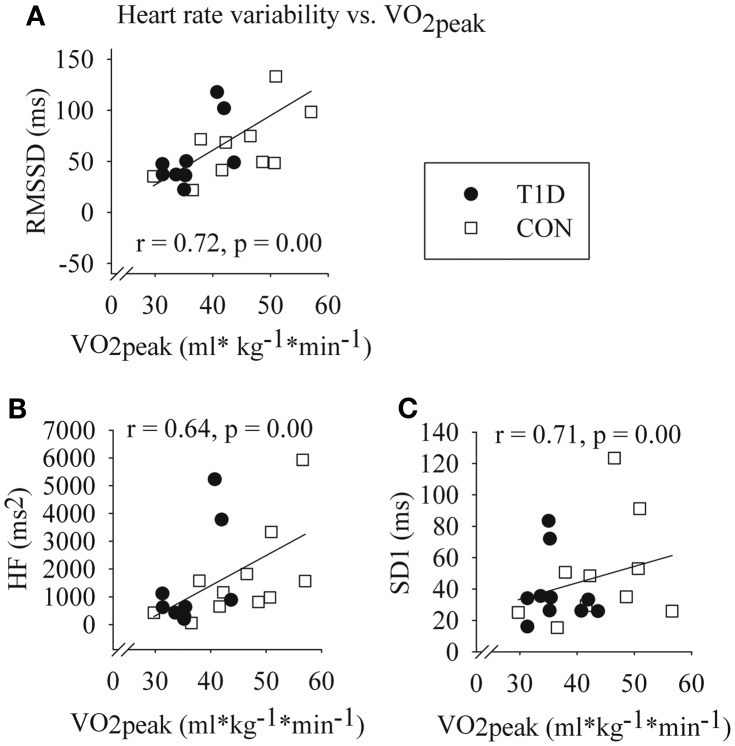
**Correlations between VO_2peak_ and square root of the mean of the sum of the squares of differences between adjacent RRIs (RMSSD) (A); high frequency component of HRV (B); and Poincaré plot variable SD1 (SD of the short-term RR interval variability) (C) at rest in type 1 diabetes patients (T1D) and in healthy controls (CON)**. Spearman correlation coefficients and *p*-values of the groups analyzed together. *n* = 10 (T1D) and *n* = 11 (CON).

The HRV measures RMSSD, HF, and SD1 at rest correlated with VO_2peak_ when the groups were analyzed together (Figure 2). The correlations remained in CON (*r* = 0.77, 0.66, and 0.73, respectively, all *p* < 0.05), but not in T1D (*r* = 0.53, 0.36, and 0.54, all *p* > 0.05). In addition, maximum to minimum ratio of deep breathing correlated to VO_2peak_ in CON (*r* = 0.77, *p* = 0.01), but not in T1D (*r* = 0.45, *p* = 0.22).

## Discussion

The main finding of this study was that T1D patients had smaller changes in non-linear detrended fluctuation analyses during active standing and handgrip than CON (Figure 1). Furthermore, in CON but not in T1D, lower VO_2peak_ was associated with lower parasympathetic cardiac activity at rest, evaluated by the HRV measures HF component, RMSSD, and SD1 (Figure 2).

Previous data have suggested functional impairment in both parasympathetic and sympathetic branches of cardiovascular ANS in T1D compared with healthy controls (Weston et al., 1998; Ziegler et al., 2001; Riihimaa et al., 2002; Vinik et al., 2003; Dalla Pozza et al., 2007; Lucini et al., 2009; Rosengård-Bärlund et al., 2009). Some studies have shown the impairments only in resting HRV, but not in the responses to active standing or the handgrip test (Lucini et al., 2009; Rosengård-Bärlund et al., 2009). We found tendencies of lower HRV median values of RMSSD, HF, and SD1 at rest in T1D compared with CON (Table 3). We did not observe differences in SBPV or BRS between T1D and CON at rest or during active standing and handgrip. However, relatively high inter- and intra-individual variability of ANS measures, together with our fairly small sample size, weakened statistical power of our results. Our T1D represented a stage of T1D where they were free of diabetes-related chronic diseases, such as cardiovascular or renal diseases, which are usually linked to T1D. This could be due to the modern efficient diagnosis and treatment of T1D, which nowadays offer good possibilities for maintaining the disease in balance, thus reducing the risks of T1D -related complications, such as impairment of ANS function.

In addition, we found differences in ANS maneuvers with non-linear detrended fluctuation analyses, as the changes of alpha1 with active standing and handgrip test were smaller in T1D than in CON (Table 3; Figure 1). Physiological background of non-linear HRV measures is not yet as well known as it is for time and frequency domain variables. Changes in alpha1 correlated with changes in the LF:HF ratio related to ANS function tests (Tulppo et al., 2001, 2005). In line with this, our results of alpha1 and LF:HF at rest and their changes related to the maneuvers were parallel (Table 3). In addition, although LF:HF changes were not significant, the median values of these changes in CON were almost twofold to those in T1D (Table 3). Alpha1 changes during active standing and in the handgrip test reflected increased cardiac sympathetic activity and a concomitant decrease in parasympathetic cardiac modulation. Tendencies of minor changes in alpha1 values with the maneuvers (Table 3; Figure 1) could indicate that T1D have attenuated cardiac sympathetic responsiveness compared with CON. Thus, although further studies are needed, non-linear HRV methods seemed to be more sensitive to detecting changes in the function of cardiac ANS related to T1D than time and frequency domain analyses. In addition, our results indicate that alongside the recordings at rest, HRV should be analyzed from recordings during maneuvers, because responsiveness is essential in managing the challenges of normal daily life.

Respiration frequency below 0.15 Hz confounds frequency domain analyses. In these cases, the respiration component of HRV may be partly or completely in the frequency area of the LF component. Although respiration frequency did not differ between T1D and CON, we removed the possible effect of slow breathing on spectral analyses by a least-means-square-based adaptive filter (Tiinanen et al., 2009). The results are not shown because the group differences in values of LF variables were similar when the data were analyzed with or without the adaptive filter, indicating that there were no significant effects of breathing frequency on the results.

Some (Niranjan et al., 1997; Komatsu et al., 2005; Gusso et al., 2008; Nadeau et al., 2010), but not all (Veves et al., 1997; Benbassat et al., 2001), studies indicate that T1D patients have lower VO_2peak_ or VO_2max_ than healthy controls. Accordingly, we recently reported lower VO_2peak_ in T1D than in CON, despite the groups were matched based on the amount of self-reported LTPA (Peltonen et al., 2012). In case of diabetic ANS dysfunction developed to a severe neuropathy, autonomic regulation of blood pressure, and heart rate during exercise could be impaired. In the present study, however, ANS function did not indicate neuropathy in T1D (Ewing et al., 1985). In addition, we recently reported similar blood pressure responses during the graded exercise test in T1D and in CON (Peltonen et al., 2012). Although beyond the scope of this study, the reasons for limited VO_2peak_ in T1D could be various and complicated, and they are not necessarily directly related to ANS function. Glycemic balance is known to affect VO_2peak_, but its mechanisms are not well known (Baldi and Hofman, 2010). Recently, we reported well-maintained arterial oxygen saturation in T1D during peak exercise, suggesting normal alveolar gas transfer (Peltonen et al., 2012). We also reported similar tissue deoxygenation in T1D and CON at peak exercise, suggesting similar mismatch between oxygen delivery and its utilization in the both study groups.

Aerobic capacity may be limited due to reduced stroke volume (Gusso et al., 2008), which in T1D is often due to diastolic dysfunction (Baldi and Hofman, 2010). Accordingly, cardiac structural and/or functional alterations possibly resulting diastolic dysfunction often exist in T1D patients, even in children and adolescents (Carugo et al., 2001; Nadeau et al., 2010; Nadeau and Reusch, 2011). Interestingly, in both type 2 diabetes (Poirier et al., 2003) and in T1D (Karamitsos et al., 2008), diastolic dysfunction has been related to cardiovascular ANS function. The mechanisms of these associations are unknown. Poirier et al. (2003) discuss that, because cardiac ANS is one regulator of myocytes’ calcium handling, could altered cardiac ANS function be a mediator of left ventricular dysfunction. The causal relation remains, however, unclear. Unfortunately, we were unable to measure cardiac left ventricular function. We recently reported, however, that earlier tissue deoxygenation in T1D than in CON during the graded exercise test along with lower VO_2peak_ suggest restrictions of cardiac output and/or peripheral vascular function in T1D (Peltonen et al., 2012).

In healthy populations, the association between enhanced vagal cardiac modulation and higher aerobic capacity is possibly linked with exercise training adaptations of the heart, such as decreased cardiac work load and oxygen demand. In the review of Hautala et al. (2009), they suggest that stronger cardiac vagal modulation could be one of the possibly mechanisms for greater training effect on VO_2max_ or VO_2peak_, and may partly explain the wide interindividual variation of training adaptation in healthy population. In our study, LTPA did not correlate with VO_2peak_ in T1D, but we found that association in CON. In addition, although not significantly, we found tendencies of lower HRV median values of RMSSD, HF, and SD1 at rest in T1D compared with CON (Table 3). The majority of these parasympathetic HRV values of T1D were situated in the lower half of the correlation plot of HRV and VO_2peak_, similar to CON with low VO_2peak_ (Figure 2). Interestingly the association between cardiac parasympathetic ANS function and VO_2peak_ was not similar in T1D and in CON. Higher parasympathetic cardiac ANS function at rest was associated with higher VO_2peak_ in CON but not in T1D (Figure 2). Also RRI response to deep breathing test did not correlate significantly with VO_2peak_ in T1D, although significant correlation existed in CON. The relative narrow distribution of VO_2peak_ in T1D may mask the phenomenon in these patients.

In the discussion of training adaptation, certain effects of training dose-response relationship on the interindividual variation of training adaptation should be also taken in to account. The dose-response relationships of training with, both aerobic capacity and ANS function are not known in T1D. The mode of training, duration, and intensity of individual exercise session, as well as training frequency and duration of training history, are known to effect on these responses in healthy populations (Uusitalo et al., 2002; Hautala et al., 2009). In addition, because of subjective nature of LTPA questionnare form, we cannot rule out the possibility of reporting bias. T1D patients might benefit from exercise training due to improvements in their cardiovascular ANS function, even in cases when their VO_2peak_ would not change significally.

Knowledge of the associations between aerobic capacity, training, and ANS function in T1D is essential. T1D is related to increased risk of cardiovascular complications (Laing et al., 2003). In general, population with a low VO_2max_ is considered to be at high risk of cardiac complications (Hooker et al., 2008; Laukkanen et al., 2010). Although the background of this association between low aerobic capacity and increased cardiac risk is not fully understood, cardiovascular autonomic regulation is one of the suggested mechanisms. This is based on evidence of the associations between altered cardiac autonomic regulation and cardiac complications, and between good cardiorespiratory fitness and potentially cardioprotective parasympathetic modulation of heart functions (Billman, 2002; Buch et al., 2002).

Physical activity is known to benefit both the subjects in increased risk of type 2 diabetes and the patients after onset of the disease. The mechanisms are probably, at least partly, improvements of insulin sensitivity and direct and/or indirect effects on cardiovascular ANS function. However, the etiology and development of type 2 diabetes and T1D are divergent, thus the study results related to exercise training and aerobic capacity cannot be generalized between these diseases. It remains to be seen in a longitudinal study whether physical exercise training has similar effects on VO_2max_ and cardiovascular ANS function in T1D patients as in type 2 diabetic or healthy populations (Buch et al., 2002; Carter et al., 2003; Bassuk and Manson, 2005; Carnethon and Craft, 2008; Hautala et al., 2009).

## Conclusion

The increase in detrended fluctuation analysis variable alpha1 during both active standing and the handgrip test was smaller in T1D than in CON, which could indicate attenuated responsiveness of sympathetic function in T1D relative to CON, although linear HRV parameters could not show this. Instead, cardiovascular ANS function at rest did not differ between T1D and CON. T1D patients had lower VO_2peak_ than healthy controls, although the groups were matched based on their self-reported LTPA. Interestingly, parasympathetic cardiac ANS variables at rest were associated with VO_2peak_ in CON, but not in T1D. The results suggest that the DFA method and the alpha1 parameter in connection with maneuvers, like active standing and handgrip, may be one of the most sensitive indicators of early ANS function changes in T1D.

## Conflict of Interest Statement

The authors declare that the research was conducted in the absence of any commercial or financial relationships that could be construed as a potential conflict of interest.
